# Trends in Access to Medications for Opioid Use Disorder

**DOI:** 10.1001/jamahealthforum.2025.0393

**Published:** 2025-04-04

**Authors:** Sumedha Gupta, Aditya James, Jennifer Miles, Hillary Samples, Stephen Crystal, Kosali Simon

**Affiliations:** 1 Department of Economics, Indiana University Indianapolis, Indianapolis; 2 Indiana University, Bloomington; 3 Center for Health Services Research, Rutgers Institute for Health, Health Care Policy and Aging Research, Rutgers University, New Brunswick, New Jersey; 4 Rutgers Robert Wood Johnson Medical School, Department of Family Medicine and Community Health, New Brunswick, New Jersey; 5 Center for Pharmacoepidemiology and Treatment Science, Rutgers Institute for Health, Health Care Policy and Aging Research, Rutgers University, New Brunswick, New Jersey; 6 Rutgers School of Public Health, Piscataway, New Jersey; 7 School of Social Work, Rutgers University, New Brunswick, New Jersey; 8 O’Neill School of Public and Environmental Affairs, Indiana University, Bloomington; 9 National Bureau of Economic Research, Cambridge, Massachusetts

## Abstract

**Importance:**

Medicaid, the largest payer for medications for opioid use disorder (MOUD), disenrolled more than 19.1 million individuals by March 2024 after the continuous coverage requirement ended in April 2023—a process termed *Medicaid unwinding*—but the impact on buprenorphine receipt remains unknown.

**Objective:**

To assess the association between Medicaid unwinding and dispensing of prescription buprenorphine, overall and by payment sources nationally and by state.

**Design, Setting, and Participants:**

Cross-sectional study of buprenorphine dispensing (age ≥18 years) from April 2020 to March 2024 using the IQVIA Longitudinal Prescription (LRx) database containing more than 90% of US retail pharmacy claims. Interrupted time-series estimated levels and trends of buprenorphine prescription dispensation before and after Medicaid unwinding.

**Main Outcomes and Measures:**

The number of patients with filled buprenorphine prescriptions each month was analyzed by payer type (Medicaid, Medicare, commercial, or self-pay) and by state. Stratified analyses assessed state factors, including automated (ex parte) Medicaid renewal rates (higher or lower than the median), income verification sources used for automated renewals (≤3, 4-5, or 6-7), and Affordable Care Act Medicaid expansion status.

**Results:**

Of the 2 405 970 adults who filled buprenorphine prescriptions between April 2020 and March 2024, 1 154 866 (48%) had at least 1 fill covered by Medicaid, 288 716 (12%) by Medicare, 1 106 746 (46%) by commercial insurance, and 264 657 (11%) by self-pay. Medicaid unwinding was associated with reversal of previously increasing trends in buprenorphine prescriptions, with 2.9% fewer patients (−23 855 [95% CI, −32 661 to −15 054]) receiving buprenorphine each month by 8 months after unwinding vs the month before unwinding began. This decline was driven by a 12.7% drop in patients with Medicaid-paid fills (−46 545 [95% CI, −51 362 to −41 730]), partially offset by increases in patients with commercial (6.12%, 19 809 [95% CI, 12 109 to 27 509]) and self-paid (7.24%, 2525 [95% CI, 1246 to 3805]) fills. Sixteen states saw overall declines in buprenorphine use after unwinding, with reductions among patients with Medicaid-covered prescriptions in 36 states, partially offset by increases in patients with commercial insurance covered fills (32 states) and self-paid fills (23 states). Buprenorphine prescriptions remained stable in states with above-median automated Medicaid renewal rates and more income verification sources, whereas states with below-median automated renewal rates, fewer verification sources, and nonexpansion state status experienced smaller offsets for Medicaid-related losses, highlighting importance of state-specific policies.

**Conclusions and Relevance:**

This cross-sectional study of Medicaid unwinding and filled buprenorphine prescriptions found that although shifts to commercial and self-pay sources mitigated some losses, rising self-pay reliance poses affordability barriers that threaten treatment continuity. Addressing access disparities is critical amid persistently high US overdose rates.

## Key Points

**Question:**

How did access to medication for opioid use disorder (MOUD) change following Medicaid’s pandemic-era enrollment freeze unwinding?

**Findings:**

In this cross-sectional study of 2.4 million US adults who filled buprenorphine prescriptions, the early unwinding period was associated with declines in the level and trend in patients receiving buprenorphine fills, driven by Medicaid-paid prescriptions, partially offset by increased commercial insurance and self-pay fills. States with higher automated (ex parte) Medicaid renewal rates and more income verification sources for automated renewals showed greater stability in buprenorphine access, highlighting the role of state policies.

**Meaning:**

Medicaid unwinding was associated with adverse reductions in buprenorphine prescriptions.

## Teaser

This cross-sectional study examines the association between Medicaid unwinding and dispensing of prescription buprenorphine, overall and by payment sources nationally and by state.

## Introduction

The Families First Coronavirus Response Act of 2020 allowed Medicaid enrollees to maintain coverage during the COVID-19 public health emergency by subsidizing states to freeze Medicaid redeterminations. From March 18, 2020, until Congress ended the freeze on March 31, 2023, Medicaid enrollment grew by nearly 23 million.^1^

How states approached unwinding the Medicaid eligibility redetermination freeze varied widely,^2,3^ reflecting states’ discretion in restarting eligibility determinations. States implemented a mix of manual and automated (ex parte) renewals that bypassed beneficiary paperwork by using relevant state or federal administrative data (eg, social welfare programs and wage or tax information) to assess eligibility.^4^ States also vary in terms of standard program operation such as administrative procedures and eligibility criteria, with those that expanded Medicaid under the Affordable Care Act (ACA) having broader eligibility. These differences in renewal policies, eligibility criteria, and administrative capacity influenced unwinding timelines. Overall, 18.7 million people nationwide lost Medicaid coverage between March 2023 and April 2024, underscoring the wide-reaching impact of unwinding.^5^

The pandemic coincided with surging rates of US drug overdose deaths, with approximately 107 543 fatalities in 2023, predominantly related to opioids.^6^ Expanding access to medication for opioid use disorder (MOUD)—including methadone, buprenorphine, and naltrexone—is a policy priority due to proven effectiveness at reducing mortality risks.^7^ As the largest payer for substance use treatment in the US,^8^ Medicaid plays a vital role in ensuring access to MOUD^9^ particularly for high-risk populations,^10^ and Medicaid expansion is associated with greater MOUD access and treatment engagement.^11^ Although the pandemic disrupted MOUD utilization growth across payers,^12,13^ Medicaid covered more MOUD prescriptions during this period than commercial insurance,^14^ partly due to enrollment growth under the eligibility freeze. Consequently, Medicaid’s unwinding could have significantly affected access to and affordability of lifesaving MOUD at a time of high need.

Given Medicaid’s critical role in MOUD access and persistently high rates of opioid overdose mortality, this study explored trends in buprenorphine receipt and payment types before and after Medicaid unwinding. We also examined whether postunwinding trends in buprenorphine use differed by state Medicaid renewal approaches (eg, automated-review rates and methods) and prepandemic Medicaid expansion status.

## Methods

This retrospective, cross-sectional study using deidentified secondary data was determined exempt from informed consent by the Indiana University institutional review board. The study followed the Strengthening the Reporting of Observational Studies in Epidemiology (STROBE) reporting guideline for cross-sectional studies.

### Data Source and Sample

We analyzed national retail pharmacy claims from April 2020 to March 2024 from the IQVIA Longitudinal Prescription (LRx) database, which tracks nearly 4 billion prescriptions dispensed in the US annually, covering more than 90% of retail pharmacy fills. Buprenorphine MOUD prescriptions were identified using National Drug Codes,^15^ excluding formulations^12^ indicated for pain management.^16^ Our final analytic sample comprised adults (≥18 years) with filled buprenorphine prescriptions across 46 states and the District of Columbia that initiated Medicaid unwinding between April and October 2023, with up to 8 months of follow-up. We excluded states that expanded Medicaid during the study period (South Dakota, Nebraska, Oklahoma, Missouri, North Carolina) to avoid conflating policy-change effects. Methadone, dispensed exclusively through federal opioid treatment programs, was excluded, as was naltrexone due to the lack of information on the underlying condition (alcohol use disorder vs OUD).

We used data from KFF (formerly the Kaiser Family Foundation) on state redetermination timelines,^1^ automated Medicaid renewals (as of October 2023),^17^ income verification sources for automated renewals (as of May 2024),^4^ and Medicaid expansion status under the ACA before the COVID-19 pandemic (eTable 1 in Supplement 1).^18^ Analyses were conducted from December 2023 to July 2024.

### Study Measures

We measured buprenorphine utilization as the number of unique patients filling any buprenorphine prescription each month, by payer type: Medicaid, Medicare Part D, commercial insurance, or cash or self-pay. We examined 3 drivers of state-level differences: (1) higher or lower than median rates of automated renewals during unwinding, (2) the number of income verification sources used for automated renewals (≤3, 4-5, 6-7), and (3) prepandemic Medicaid expansion status.

### Statistical Analysis

We analyzed national- and state-level trends in buprenorphine prescription dispensing before and after Medicaid unwinding using interrupted time-series (ITS) models,^19^ centered on the month each state initiated unwinding (see eAppendix A in Supplement 1 for details). Models included indicators for the unwinding period (before and after unwinding intercept or level), continuous month since the start of the study period (preunwinding trend), and a continuous month since the start of the unwinding (postunwinding trend). A significant difference in the before and after unwinding coefficient indicates immediate (intercept or level) shifts in buprenorphine receipt, whereas differences in before and after unwinding trends reflect changes in growth rate over time associated with unwinding.

We estimated unwinding-associated changes in monthly buprenorphine receipt, overall and by payer type and state. To assess how state Medicaid administration influenced these changes, we stratified analyses by higher or lower than median automated renewal rates, income verification sources (≤3, 4-5, 6-7), and prepandemic Medicaid expansion status.

To summarize buprenorphine dispensing during the 36-month preunwinding period, we used ITS coefficients. We first calculated the difference between regression-adjusted estimates in the last month before unwinding (month −1) and the first study month (month −36), overall and by payer. Then, we calculated the percent change in dispensing by dividing the change in estimates by the baseline estimate (month −36) and multiplying by 100. These relative percent change statistics summarize the improvement in MOUD utilization during the preunwinding period, overall, by payer, and by state.

We used ITS coefficients to estimate changes in buprenorphine dispensing through the final study month (month 8) after unwinding, calculating the sum of the immediate level change, and 8 times the monthly trend change. To contextualize the postunwinding change, we present the percent change in month 8 after unwinding, relative to the month prior to unwinding (month −1) at national, state, and state-group levels, overall and by payer. Additionally, to compare with national estimates, we also presented ITS-based, 8-month postunwinding relative percent changes in state samples stratified by higher or lower than median automated Medicaid renewal rates, income verification sources, and prepandemic Medicaid expansion status.

All analyses were conducted using Stata version 18.1 (StataCorp),^20^ with 2-sided *t* tests and significance set at *P* < .05.

## Results

### Sample Characteristics

Our sample included 2 405 970 adults who filled buprenorphine prescriptions between April 2020 and March 2024. Among people with buprenorphine fills, 1 154 866 (48%) had at least 1 fill covered by Medicaid, 288 716 (12%) by Medicare, 1 106 746 (46%) by commercial insurance, and 264 657 (11%) by self-pay (individuals could have multiple payment sources). eTable 2 in Supplement 1 presents sample sizes by payer and year. The percentage of adults with buprenorphine fills paid by Medicaid increased from 46% (April 2020-March 2021) to 49% (April 2023-March 2024).

Four states initiated redeterminations in April, 14 in May, 22 in June, 9 in July, and 1 in October. As of October 2, 2023, an average of 55.9% of individuals who retained Medicaid coverage after the resumption of redeterminations were renewed through automated processes across states (median, 62.97%; IQR, 36.82%-75.91%). However, the rates of automated renewals varied widely, ranging from 2% in Wyoming to 99% in North Carolina. The number of data sources used to verify income also varied; 14 states used fewer than 3 sources; 27 states, 4 to 5 sources; and 9 states, 6 to 7 sources of income data to verify Medicaid beneficiary income eligibility. By the onset of the COVID-19 public health emergency, 36 states had already expanded Medicaid, and 10 states had not. See eTable 1 in Supplement 1 for state-level measures.

### Buprenorphine Receipt Prior to Medicaid Unwinding

**Figure 1.**
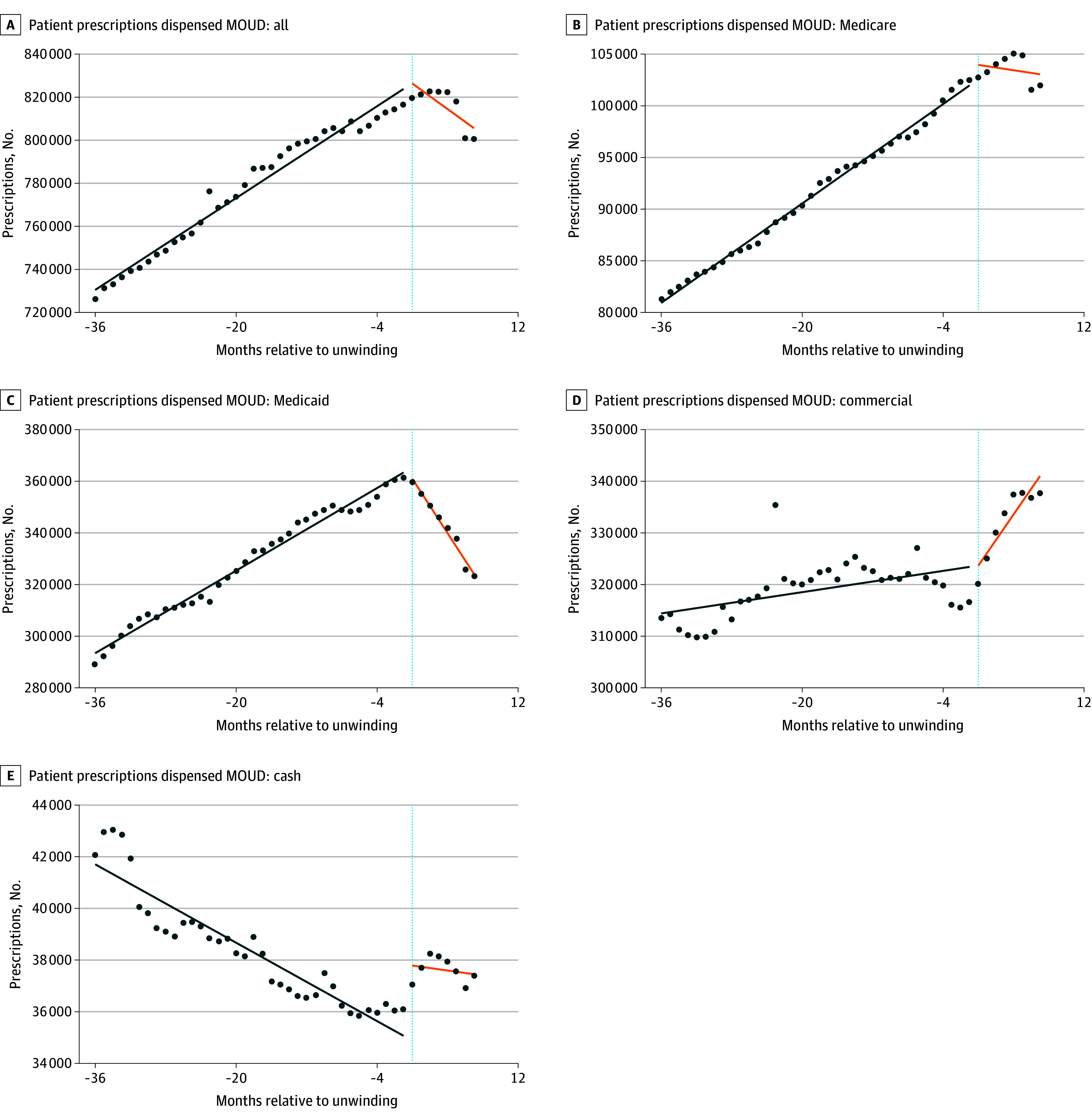
Estimated Monthly Patients With Buprenorphine Prescription Fills by Payer Type The estimated number of US adults, 18 years or older, with filled buprenorphine prescriptions each month between April 2020 to March 2024 are presented. All models use data from 45 US states and Washington, DC; 5 states that expanded Medicaid under the Affordable Care Act during the study period—South Dakota, Nebraska, Oklahoma, Missouri, and North Carolina—were excluded from the analysis. Data are centered at the time of the start of Medicaid unwinding in each state, and 36 months prior to the start of the unwinding and 8 months after unwinding are examined for all states. The lines indicate the interrupted time series model–based estimates of the number of patients with buprenorphine fills before (green) and after (orange) the unwinding. MOUD indicates medication for opioid use disorder.

In the first month of the preunwinding period (month −36), 752 472 patients filled buprenorphine prescriptions. Among them, 338 612 (45%) had at least 1 fill paid by Medicaid, 82 772 (11%) by Medicare, 353 662 (47%) by commercial insurance, and 90 297 (12%) by self-pay. During the preunwinding period, the estimated number of patients with buprenorphine fills steadily increased by 2668 (95% CI, 2502 to 2834) patients each month (preunwinding trend in the Table and Figure 1), driven by substantial increases among patients with fills paid by Medicaid each month (1999 [95% CI, 1908 to 2090]), with modest increases among patients with fills paid each month by both commercial insurance (257 [95% CI, 112 to 403]) and Medicare (601 [95% CI, 578 to 624]). As access to buprenorphine insurance coverage increased, patients with self-paid buprenorphine fills each month declined by −190 (95% CI, −214 to −165).

**Table.** Changes in Monthly Number of Patients With Filled Buprenorphine Prescriptions

Variable	Patients with filled prescriptions, No. (95% CI)				
	Total	Medicaid	Medicare	Commercial	Self-pay
**Estimated intercept^a^**					
Unwinding period					
Before	826 466 (822 942 to 829 990)	365 352 (363 424 to 367 279)	102 569 (102 080 to 103 057)	323 667 (320 585 to 326 750)	34 879 (34 366 to 35 391)
After	826 323 (819 640 to 833 006)	360 911 (357 255 to 364 567)	103 956 (103 030 to 104 882)	323 668 (317 822 to 329 513)	37 788 (36 816 to 38 759)
Before-to-after change in level (ie, intercept)^b^	−144 (−7700 to 7412)	−4400 (−8600 to −307)	1387 (340 to 2434)	0 (−6600 to 6609)	2909 (1811 to 4007)
*P* value	.97	.04	.013	>.99	.003
**Estimated monthly trend (slope)**					
Unwinding period					
Before	2668 (2502 to 2834)	1999 (1908 to 2090)	601 (578 to 624)	257 (112 to 403)	−190 (−214 to −165)
After	−2964 (−4562 to −1367)	−5263 (−6137 to −4389)	−129 (−350 to 92)	2476 (1079 to 3873)	−48 (−280 to 184)
Before-to-after change in trend (ie, slope)^b^	−5600 (−7200 to −4000)	−7300 (−8100 to −6400)	−730 (−953 to −508)	2219 (814 to 3623)	142 (−92 to 375)
*P* value	<.01	<.01	<.01	.003	.23
Effect after 8 mo after unwinding^c^	−23 855 (−32 661 to −15 054)	−46 545 (−51 362 to −41 730)	355 (−866 to 1573)	19 809 (12 109 to 27 509)	2525 (1246 to 3805)
Relative percent change after 8 mo^d^	−2.89	−12.74	0.35	6.12	7.24
*P* value	<.001	<.001	.56	<.001	<.005

^a^ Estimates were calculated from ordinary least-squares regressions of the monthly number of patients with filled buprenorphine prescriptions for opioid use disorder on monthly trends (interrupted time-series analyses).

^b^ Differences in intercepts and slopes and their 95% CIs were used to test before-to-after change.

^c^ The prescription dispensing outcome in month 8 after unwinding was calculated by summing the postunwinding intercept and 8 times the postunwinding monthly trend (eg, total number of patients with filled buprenorphine prescriptions in month 8 after unwinding: 826 323 + 8 × −2964 = 802 611). Then, the 8-month postunwinding effect was calculated as the difference between the estimated dispensing outcome in month 8 after unwinding and the preunwinding level (level-intercept), ie, 802 611 −826 466 = −23 855.

^d^ The relative percent change after 8 months was calculated by dividing the estimated 8-month change by the preunwinding level (intercept), and multiplying by 100 (eg, relative percent change in the number of patients with filled buprenorphine prescriptions 8 months after unwinding = (−23 855/826 466) × 100 = −2.89).

Over the 3-year preunwinding period, the number of individuals receiving buprenorphine fills each month increased by 9.8%, rising from the first (month −36) to the final month (month −1), to reach 826 466 (95% CI, 822 942–829 990). This overall increase was driven by increases of patients each month with Medicaid fills (365 352 [95% CI, 363 424-367 279]; 22.9%), commercial fills (323 667 [95% CI, 320 585-326 750]; 2.5%), and Medicare fills (102 569 [95% CI, 102 080-103 057]; 20.2%). Additionally, the percentage of patients who self-paid each month for buprenorphine declined by 26.8% during this period (34 879 [95% CI, 34 366-35 391]). Thus, during the preunwinding period, Medicaid became the primary payer for patients with buprenorphine fills.

### Changes in Buprenorphine Receipt Associated With Medicaid Unwinding

Following Medicaid unwinding, there was an immediate level or intercept decline of 4400 (95% CI, −8600 to −307) patients with buprenorphine prescriptions paid by Medicaid, which was offset by increases in the number of patients whose monthly fills were paid by Medicare (1387; 95% CI, 340-2434) or were self-paid (2909; 95% CI, 1811-4007) (Table).

Medicaid unwinding was also associated with a monthly trend decline of −5600 (95% CI, −7200 to −4000) patients receiving buprenorphine prescriptions, driven by a large reduction for Medicaid (−7300 [95% CI, −8100 to −6400]) and a smaller decline for Medicare (−730 [95% CI, −953 to −508]). In contrast, there was an increasing trend for commercial insurance (2219 [95% CI, 814 to 3623]).

Our ITS estimates showed that 8 months after the start of Medicaid unwinding (month 8), there were 23 855 (95% CI, −32 661 to −15 054) fewer patients receiving buprenorphine nationwide, representing a statistically significant 2.89% reduction (*P* < .001) compared with the month immediately preceding unwinding (month −1) (Table and eFigure 1 in Supplement 1). This decline was primarily driven by a 12.74% reduction (−46 545 [95% CI, −51 362 to −41 730]; *P* < .001) in patients with fills paid by Medicaid, despite a 6.12% increase (19 809 [95% CI, 12 109 to 27 509]; *P* < .001) in patients with fills paid by commercial insurance and a 7.24% increase (2525 [95% CI, 1246 to 3805]; *P* < .005) in patients with self-paid fills. There was no significant change in patients whose fills were paid by Medicare.

### State Variations

Both eFigure 2 and eTable 3 in Supplement 1 highlight significant state variation in growth of patients with buprenorphine fills each month during the preunwinding period by payer type, predominantly driven by prescriptions paid by Medicaid (increases of 0%-50% in 32 states, 50%-100% in 4 states, >100% in 6 states).

**Figure 2.**
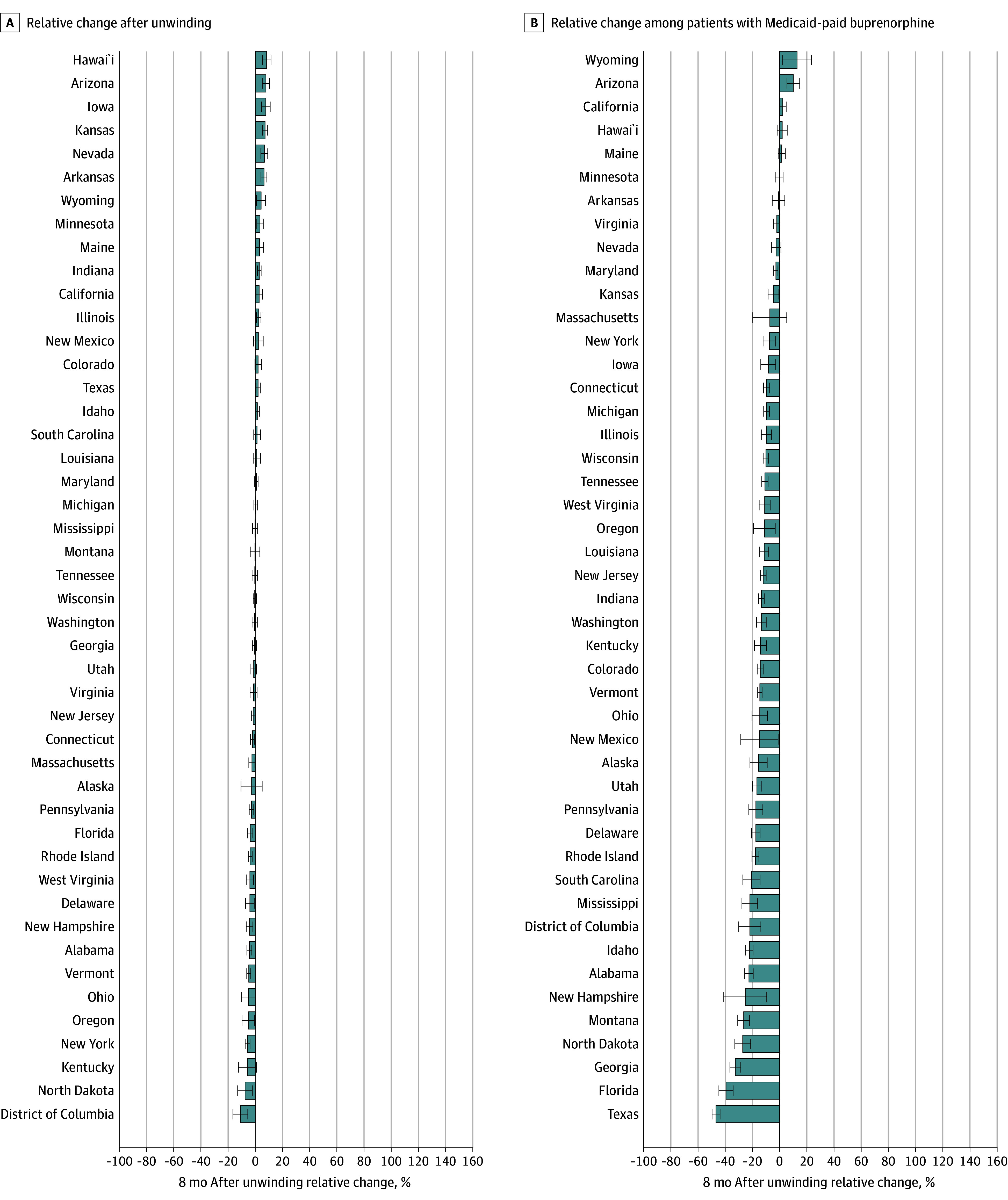
State Changes in Monthly Number of Patients With Filled Buprenorphine Prescriptions, Overall and by Medicaid An interrupted time-series analysis, covering 36 months before and 8 months after the start of Medicaid unwinding (April 2020-March 2024) in each state, was used to evaluate percent changes in the number of adults (age >18 years) with filled buprenorphine prescriptions during the 8 months following the unwinding in each state. The analysis considered both the overall number of prescriptions and those paid by Medicaid, using data from 45 US states and Washington, DC. Five states that expanded Medicaid during the study period were excluded. (See the legend in Figure 1 for the excluded states.) The relative percent change in patients with buprenorphine fills each month after 8 months after unwinding was calculated by dividing the estimated change (sum of the postunwinding intercept and 8 times the postunwinding monthly trend relative to the preunwinding intercept) by the preunwinding intercept, then multiplying by 100. The bars represent the interrupted time series model–based estimates of the percentage change in the number of patients with buprenorphine fills 8 months after the unwinding relative to the preunwinding period; the whiskers, 95% CIs.

**Figure 3.**
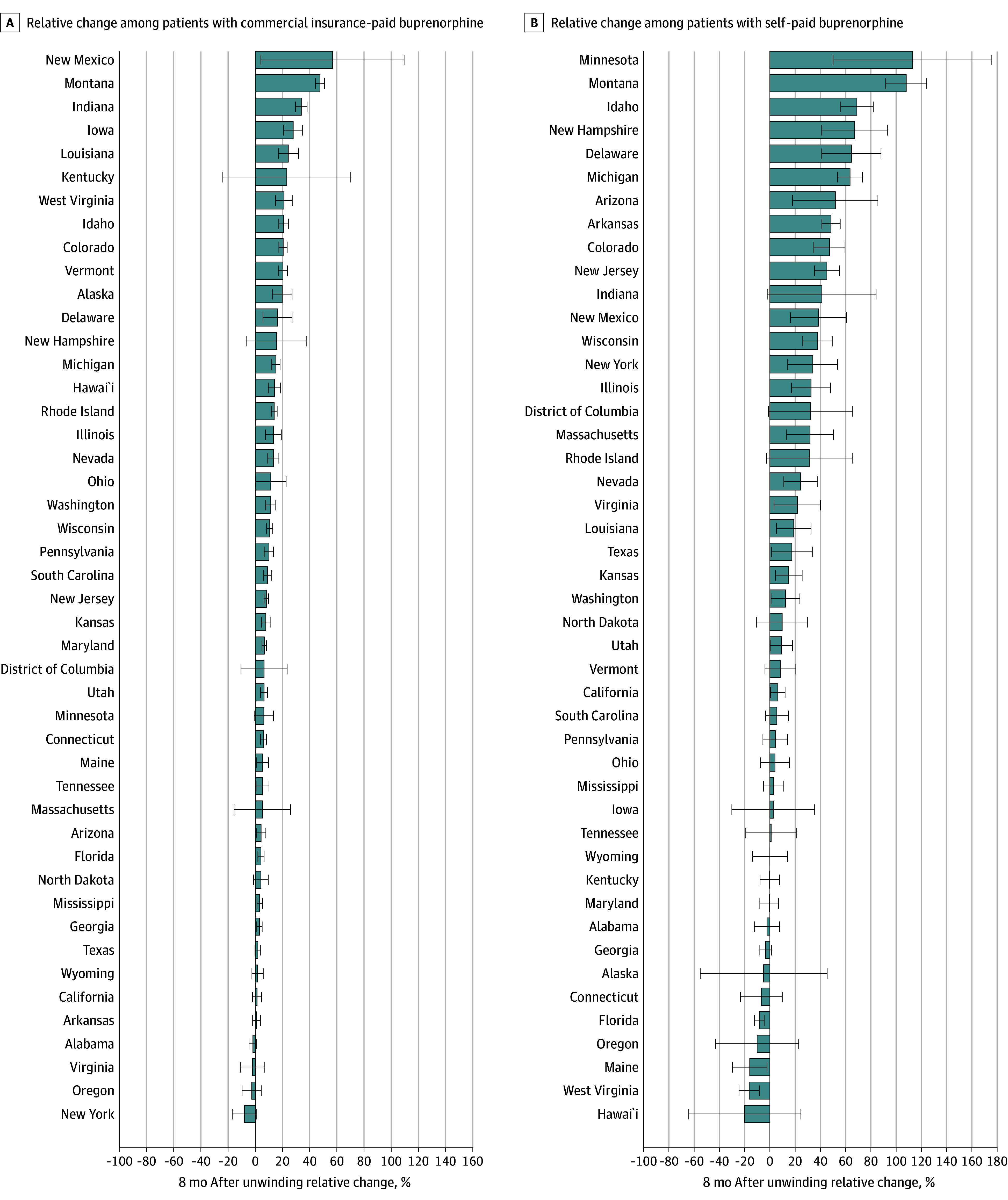
State Changes in Monthly Number of Patients With Filled Buprenorphine Prescriptions, Paid by Commercial Insurance and Self-Paid An interrupted time-series analysis, covering 36 months before and 8 months after the start of Medicaid unwinding (April 2020-March 2024) in each state, was used to evaluate percent changes in the number of adults (age >18 years) with filled buprenorphine prescriptions during the 8 months following the unwinding in each state. The analysis considered both the overall number of prescriptions and those paid by Medicaid, using data from 45 US states and Washington, DC. Five states that expanded Medicaid during the study period were excluded. (See the legend in Figure 1 for the excluded states.) The relative percent change in patients with buprenorphine fills each month after 8 months after unwinding was calculated by dividing the estimated change (sum of the postunwinding intercept and 8 times the postunwinding monthly trend relative to the preunwinding intercept) by the preunwinding intercept, then multiplying by 100. The bars represent the interrupted time series model–based estimates of the percentage change in the number of patients with buprenorphine fills 8 months after the unwinding relative to the preunwinding period; the whiskers, 95% CIs.

State-specific ITS models revealed significant variation in change in the number of patients receiving buprenorphine each month (Figure 2 and eTable 4 in Supplement 1). Compared with the month immediately preceding Medicaid unwinding (month −1), the number of patients with filled buprenorphine prescriptions in month 8 after unwinding was significantly lower in 16 states (Figure 2A). This overall decline was primarily driven by reductions for Medicaid in 36 states (Figure 2B), with compensatory increases for commercial insurance in 32 states (Figure 3A) and for self-pay in 23 states (Figure 3B).

**Figure 4.**
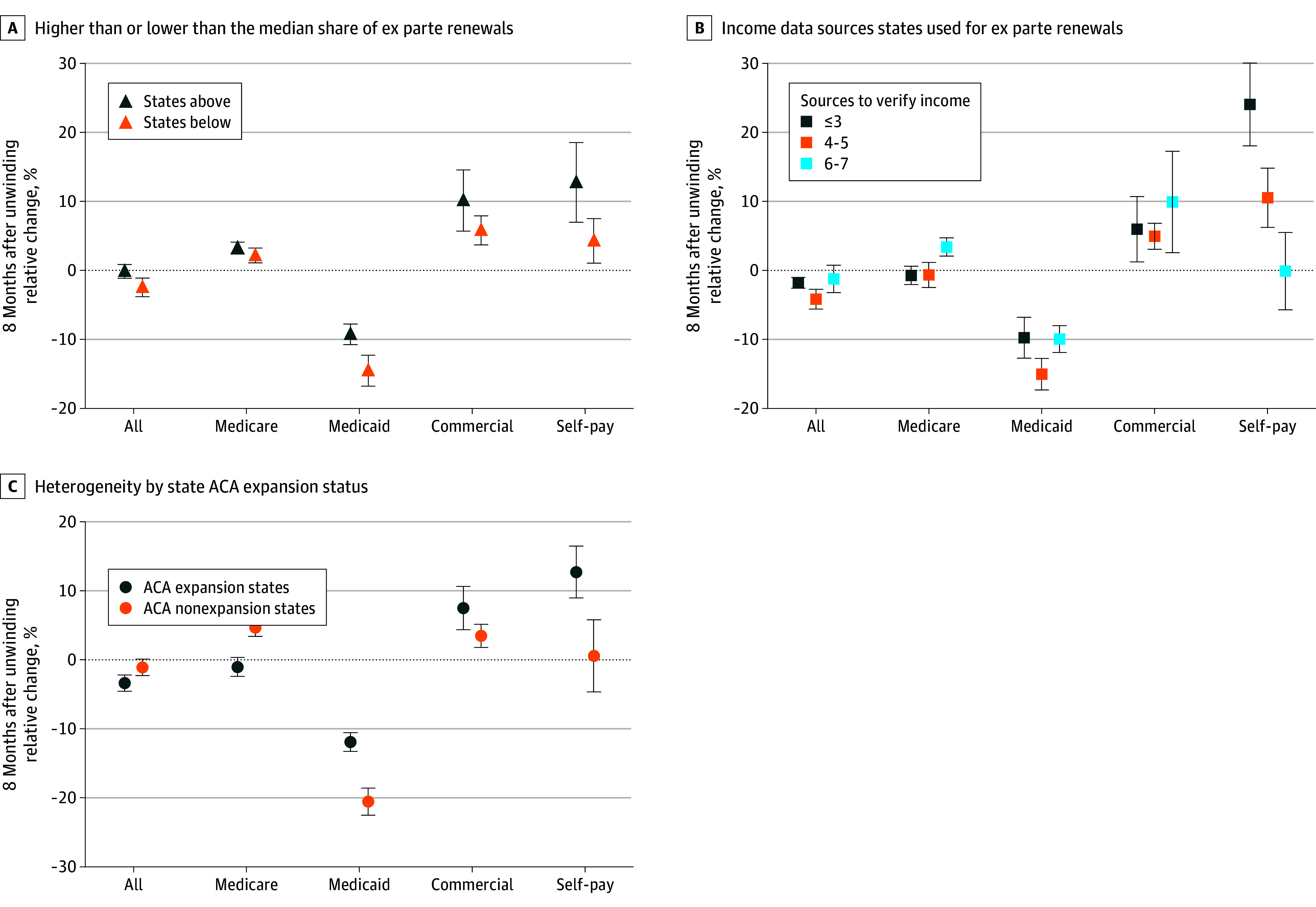
State Changes in Monthly Number of Patients With Filled Buprenorphine Prescriptions, Overall, and by Payer An interrupted time-series analysis, covering 36 months before and 8 months after the start of Medicaid unwinding (April 2020-March 2024) in each state, was used to evaluate percent changes in the number of adults (age >18 years) with filled buprenorphine prescriptions during the 8 months following the unwinding in each state. The analysis considered both the overall number of prescriptions and those paid by Medicaid, using data from 45 US states and Washington, DC. Five states that expanded Medicaid during the study period were excluded. (See the legend in Figure 1 for the excluded states.) The relative percent change in patients with buprenorphine fills each month after 8 months after unwinding was calculated by dividing the estimated change (sum of the postunwinding intercept and 8 times the postunwinding monthly trend relative to the preunwinding intercept) by the preunwinding intercept, then multiplying by 100. The data points represent the interrupted time series model–based estimates of the percentage change in the number of patients with buprenorphine fills 8 months after the unwinding relative to the preunwinding period; the whiskers, 95% CIs. ACA indicates Affordable Care Act.

Stratified analysis of states higher or lower than the median automated renewal rates (Figure 4; eTable 5 in Supplement 1) revealed that the decline in patients with buprenorphine fills in month 8 after unwinding (−2.46% [95% CI, −3.82% to −1.11%]) relative to the month prior to the start of unwinding (month −1) was concentrated in states with rates lower than the median. States with lower than the median rates also showed larger reductions in patients with buprenorphine fills paid by Medicaid (−14.54% [95% CI, −16.78% to −12.30%]) than in states higher than the median (−9.26% [95% CI, −10.75% to −7.77%]). Similarly, there were small increases in states lower than the median vs those higher than the median for Medicare (2.14% [95% CI, 1.06% to 3.21%] vs 3.23% [95% CI, 2.38% to 4.09%]), commercial insurance (5.80% [95% CI, 3.70% to 7.90%] vs 10.11% [95% CI, 5.69% to 14.52%]), and self-pay (4.27% [95% CI, 1.03% to 7.51%] vs 12.73% [95% CI, 6.97% to 18.49%]), respectively.

States using the most data sources for income verification in automated renewals (6-7) showed no overall decline in buprenorphine receipt (eTable 5 in Supplement 1) due to reductions in fills paid by Medicaid (−9.94% [95% CI, −11.88% to −8.00%]) being offset by larger increases of fills paid by Medicare (3.40% [95% CI, 2.08% to 4.73%]; no significant change for ≤3 or 4–5 income data sources) and by commercial insurance (9.90% [95% CI, 2.55% to 17.25%]). In contrast, states using 3 or fewer or 4 to 5 data sources experienced similar declines in Medicaid-paid fills (≤3, −9.74% [95% CI, −12.71% to −6.78%]; 4–5, −15.05% [95% CI, −17.33% to −12.77%]), but were not fully offset by the smaller increases in commercial insurance fills (≤3, 5.94% [95% CI, 1.20% to 10.69%); 4-5, 4.94% [95% CI, 3.05% to 6.82%]) and despite larger increases in self-paid fills (24.07% [95% CI, 18.04% to 30.11%] and 10.53% [95% CI, 6.23% to 14.83%], respectively). Fills paid by Medicare showed no significant changes in states using fewer than 6 data sources.

Overall changes in patients with buprenorphine fills by month 8 after unwinding compared with the month preceding unwinding were similar in magnitude nationwide (−2.89% [95% CI, −3.95% to −1.82%]) and in Medicaid expansion states (−3.40% [95% CI, −4.56% to −2.23%]) (eTable 5 in Supplement 1). Significant declines in Medicaid-paid fills were observed in both expansion (−11.93% [95% CI, −13.29% to −10.57%]) and nonexpansion states (−20.56% [95% CI, −22.52% to −18.60%]). However, offsets differed, with smaller increases in nonexpansion vs expansion states in the percentage of patients whose buprenorphine was paid by commercial insurance (3.47% [95% CI, 1.82% to 5.11%] vs 7.47% [95% CI, 4.34% to 10.59%]) and the percentage of patients who self-paid (no increase vs 12.70% [95% CI, 8.96% to 16.45%]), respectively, but larger increases in Medicare (4.65% [95% CI, 3.40% to 5.91%]) in nonexpansion states vs no change in Medicare covered prescriptions in expansion states.

## Discussion

This cross-sectional study provides new insights into how Medicaid unwinding was associated with reduced access to buprenorphine during a critical phase of the opioid epidemic. As in prior studies, a continued but slowed increase in buprenorphine use during the COVID-19 pandemic was found,^12,13^ with increases primarily driven by prescriptions paid by Medicaid. This trend likely reflects the suspension of Medicaid eligibility redeterminations alongside policy-driven expansions in MOUD access that were national in scope, such as the March 2020 federal authorization of telemedicine-based buprenorphine inductions.^21,22^

By the end of the study period, the nationwide number of patients filling buprenorphine prescriptions had declined compared with the month before Medicaid redeterminations resumed, driven by reductions in patients with buprenorphine prescriptions paid by Medicaid. About half of the Medicaid decrease was not offset by other payment sources, whereas the rest was offset by increases in the number of patients whose prescriptions were paid by commercial insurance or who self-paid for them. A recent study highlighted 2 potential key explanations for the rise in commercial insurance-paid MOUD after unwinding.^23^ First, about half of people losing Medicaid coverage were enrolled in employer-sponsored insurance, and simultaneous Medicaid and private insurance coverage during the pandemic^24,25^ may have enabled an immediate transition to commercial coverage. Second, marketplace enrollment likely increased due to the Medicaid unwinding special enrollment period and enhanced subsidies from the American Rescue Plan.^26,27,28,29^ However, higher cost sharing under commercial insurance may reduce affordability, access, and medication stability compared with Medicaid’s typically lower out-of-pocket costs.^30^ Higher cost-sharing burdens are associated with shorter buprenorphine treatment retention,^31,32^ with one study reporting a 34% higher likelihood of discontinuation for patients in the highest vs lowest cost-sharing quartile.^33^ Patients paying fully out of pocket face even greater financial barriers to consistent use.^30^ Even those maintaining MOUD access after unwinding may experience coverage disruptions, leading to long-term declines in buprenorphine use and related harms. Further research is needed to understand price sensitivity in the Medicaid population.

Significant state-level variation in buprenorphine receipt changes following Medicaid unwinding was also observed, possibly driven by state-specific renewal strategies and preexisting policies like Medicaid expansion. This underscores the decentralized nature of Medicaid and the critical role of state administration in shaping access to buprenorphine MOUD. States like Texas, Florida, and Georgia experienced the largest declines in Medicaid-paid buprenorphine use, influenced by high disenrollment rates (eg, 2.5 million in Texas as of August 2024),^5^ often due to procedural issues (eg, 78% of disenrollments in Georgia), resulting in litigation (eg, Florida).^34^ In contrast, states like California mitigated declines with automatic renewals and the removal of asset tests.^35,36^ Administrative flexibility played a key role. For example, Arkansas completed renewals in 6 months, whereas Kentucky and Louisiana took 12 months; Kentucky paused redeterminations for children; and Texas prioritized cases that were likely ineligible.^37^ These variations highlight the need for ongoing monitoring of unwinding impacts on OUD treatment.

### Limitations

This study has several limitations. First, although the data only include retail pharmacy claims and excludes buprenorphine dispensed in opioid treatment program settings, the dataset accounts for more than 15 million prescriptions in 2023, highlighting the relevance of examining Medicaid unwinding-associated changes in this context. Relatedly, not captured was methadone dispensed from opioid treatment programs, which commercial insurers rarely cover and may have steeper declines after unwinding. Second, this study could not distinguish between commercial insurance obtained through employer-sponsored plans and marketplace coverage, limiting insights into specific sources of increased commercial coverage. Additionally, this study captured payer type at the prescription level, restricting analysis of patients with multiple insurance types (eg, dual Medicaid-Medicare coverage). Third, the lack of race and ethnicity data prevents analysis of disparities in buprenorphine access that may have disproportionately impacted minoritized populations. Similarly, the absence of clinical data (eg, diagnoses) limits understanding for groups with greater medical and behavioral health needs. Fourth, unmeasured policy factors, such as relaxed requirements enabling telemedicine-based buprenorphine inductions (March 2020)^21^ and eliminations of the 30-patient waiver (April 2021) and X-waiver (December 2022)^38^ may also influence buprenorphine access. Evidence suggests that telehealth-enabled inductions expanded buprenorphine use,^22^ but the waiver changes did not.^14,39,40,41,42^ Because these policies were implemented at the national level, and not specific to Medicaid, they are unlikely to confound our estimates. Fifth, the near-simultaneous unwinding across states precludes a counterfactual and thereby causal interpretation of the findings. Additionally, ITS models were linear regressions. Although some subgroups may have nonlinear trends, limited postperiod data prevented the use of more complex models, which are suggested for future studies.^43,44^ Also, overlapping confidence intervals indicate state-level variation but preclude formal statistical testing of the association between policy differences and buprenorphine access after unwinding. Sixth, the focus on buprenorphine MOUD excludes potential changes in other medication classes, warranting further research.

## Conclusions

Although short-term reductions in buprenorphine use associated with Medicaid unwinding were modest on average in this study, future research is needed to assess long-term effects of Medicaid unwinding on efforts to expand evidence-based care to tackle the overdose crisis. Continued evaluation is essential to understand state-level variations and the policy implications of differing Medicaid renewal strategies and eligibility policies on broader health care access.

## Notes

Supplement 1.
eAppendix
**eTable 1.** Timeline of state Medicaid unwinding, rates and approaches to automated renewals, and state Medicaid Affordable Care Act (ACA) expansion status **eTable 2.** Study sample size by year and payer type **eTable 3.** Estimated change in Monthly Patients with Buprenorphine during the 3-year period prior to Medicaid unwinding by Payer Type **eTable 4.** State Changes in Monthly Number of Patients with filled Buprenorphine Prescriptions by payer type **eTable 5.** State Changes in Monthly Number of Patients with filled Buprenorphine Prescriptions by payer type and state policies that may support continued coverage for eligible individuals **eFigure 1.** Changes in Monthly Number of Patients with filled Buprenorphine Prescriptions, overall and prescription fills paid for by Medicaid **eFigure 2.** Estimated change in Monthly Patients with Buprenorphine Prescription Fills during the 3-year period prior to Medicaid unwinding by Payer Type
eReferences

Supplement 2.
Data Sharing Statement
