# Supplementary material for: Trends in Access to Medications for Opioid Use Disorder
Source: JAMA Health Forum. 2025 Apr 4;6(4):e250393. doi: 10.1001/jamahealthforum.2025.0393 (PMC11971676; doi:10.1001/jamahealthforum.2025.0393)
Supplement: Supplement 1. — eAppendix eTable 1. Timeline of state Medicaid unwinding, rates and approaches to automated renewals, and state Medicaid Affordable Care Act (ACA) expansion status eTable 2. Study sample size by year and payer type eTable 3. Estimated change in Monthly Patients with Buprenorphine during the 3-year period prior to Medicaid unwinding by Payer Type eTable 4. State Changes in Monthly Number of Patients with filled Buprenorphine Prescriptions by payer type eTable 5. State Changes in Monthly Number of Patients with filled Buprenorphine Prescriptions by payer type and state policies that may support continued coverage for eligible individuals eFigure 1. Changes in Monthly Number of Patients with filled Buprenorphine Prescriptions, overall and prescription fills paid for by Medicaid eFigure 2. Estimated change in Monthly Patients with Buprenorphine Prescription Fills during the 3-year period prior to Medicaid unwinding by Payer Type eReferences [file jamahealthforum-e250393-s001.pdf]

Supplemental Online Content

Gupta S, James A, Miles J, Samples H, Crystal S, Simon K. Trends in access to medications for opioid use disorder. *JAMA Health Forum*. doi:10.1001/jamahealthforum.2025.0393

eAppendix

eTable 1. Timeline of state Medicaid unwinding, rates and approaches to automated renewals, and state Medicaid Affordable Care Act (ACA) expansion status

eTable 2. Study sample size by year and payer type

eTable 3. Estimated change in Monthly Patients with Buprenorphine during the 3-year period prior to Medicaid unwinding by Payer Type

eTable 4. State Changes in Monthly Number of Patients with filled Buprenorphine Prescriptions by payer type

eTable 5. State Changes in Monthly Number of Patients with filled Buprenorphine Prescriptions by payer type and state policies that may support continued coverage for eligible individuals

eFigure 1. Changes in Monthly Number of Patients with filled Buprenorphine Prescriptions, overall and prescription fills paid for by Medicaid

eFigure 2. Estimated change in Monthly Patients with Buprenorphine Prescription Fills during the 3-year period prior to Medicaid unwinding by Payer Type

eReferences

This supplemental material has been provided by the authors to give readers additional information about their work.

© 2025 Gupta S, et al. *JAMA Health Forum*.

eAppendix A. Description of the Interrupted Times Series Regression Approach

The analysis evaluated changes in levels and trends of patients using buprenorphine for medication for opioid use disorder (MOUD), both overall and by payer, associated with the unwinding of the COVID-19 public health emergency (PHE) Medicaid redetermination suspension. To estimate nationwide changes (denoted state grouping $G = n$), we utilized national- month relative to unwinding level data on the number of patients using buprenorphine MOUD, spanning 36 months prior to and 8 months post unwind (denoted $t \in [-36,8]$) level data. This was done by first centering all state-specific observations relative to the timing of the unwinding in each state and subsequently aggregating the data to the national level, weighted by the annual state population from the U.S. Census,[1] both overall and stratified by payer type $p \in \{All, Medicare, Medicaid, Commercial, Self - Pay\}$. For state-specific estimates ($G = s;\ s = 1 \dots S$), we analyzed state-month relative to unwinding level data from the same time frame ($t \in [-36,8]$) to assess changes associated with each state's Medicaid unwinding activities, again both overall and by payer.

Additionally, we explored three potential drivers of state-level variations in post-unwinding outcomes by re-estimating regression models using aggregated monthly data for stratified state samples:

1. States with above- or below-median rates of automated (*ex parte*) Medicaid eligibility renewals during the unwinding ($ExparteRate \in ExparteAboveMedian, ExparteBelowMedian\}$).
2. States grouped by the number of data sources used for income verification in ex parte renewals ($IncomeVerSources \in \{\leq 3, 4\text{–}5, \text{or } 6\text{–}7\}$).

© 2025 Gupta S, et al. *JAMA Health Forum*.

3. States that had implemented Medicaid expansion under the ACA prior to the COVID-19 pandemic versus those that had not ($ACAExpStatus \in \{ACAExp, ACANonExp\}$).

Thus, $G \in \{n, s, ExparteRate, IncomeVerSources, ACAStatus\}$ represents the different stratified groups considered in the analysis.

Model of number of patients using buprenorphine MOUD For estimates of the changes in the number of patients using buprenorphine MOUD associated with the unwinding we estimated regressions of the following form:

$$Y_{G,t}^{p} = \beta_{G,0}^{p} PreI_{G,t}^{p} + \beta_{G,1}^{p} PreT_{G,t}^{p} + \beta_{G,2}^{p} PostI_{G,t}^{p} + \beta_{G,3}^{p} PostT_{G,t}^{p} + \varepsilon_{G,t}^{p} \quad (1)$$

Where $Y_{G,t}^{p}$ represents the dependent variable the number of patients using buprenorphine MOUD paid for by payer p, in sample G, in event-month t. On the right-hand side, $PreI_{G,t}^{p}$ is a binary indicator for the pre-unwinding period (pre- unwinding $PreI_{G,t}^{p} = 1$, post-unwinding $PreI_{G,t}^{p} = 0$) with $\beta_{G,0}^{p}$ capturing the level/intercept of the dependent variable in the month immediately preceding the start of unwinding (i.e., $t = -1$) for payer p and sample G. $PreT_{G,t}^{p}$ represents the time elapsed since start of the study period (i.e., sequentially takes values from -36 to -1, starting in the first month of the pre-unwinding period (month -36) and ending in the final month before the unwinding began (month -1), with value of 0 in all post-unwinding periods) with $\beta_{G,1}^{p}$ capturing the pre-unwinding trend in the dependent variable. $PostI_{G,t}^{p}$ is a binary indicator variable representing the post-unwinding period (pre- unwinding $PostI_{G,t}^{p} = 0$, post-unwinding $PostI_{G,t}^{p} = 1$), with $\beta_{G,2}^{p}$ capturing the post-unwinding level/intercept of the dependent variable. $PostT_{G,t}^{p}$ is the time since start of unwinding (value of 0 in all pre-unwinding periods, with successive values between 0 and 8, in each month since start of the unwinding), and $\beta_{G,3}^{p}$ captures the post-unwinding trend in the dependent variable. All analysis used heteroskedasticity robust standard errors. The difference in the estimated intercept coefficients ($\widehat{\beta_{G,2}^{p}} - \widehat{\beta_{G,0}^{p}}$) is the change in the level of the dependent variable for state sample group G and payer type p following unwinding and difference in the estimated slope coefficients ($\widehat{\beta_{G,3}^{p}} - \widehat{\beta_{G,1}^{p}}$) is the change in the nationwide trend of the dependent variable for group G and payer type p following unwinding.

© 2025 Gupta S, et al. *JAMA Health Forum*.

For all national and stratified state samples, the prescription dispensing outcome 8-months post-unwinding was calculated by summing the post-unwinding intercept and eight times the post-unwinding monthly trend. Then, the 8-months post-unwinding effect was calculated as the difference between the estimated dispensing outcome 8-months post-unwinding and the pre-unwinding level (intercept). Finally, the relative percent change over 8 months was calculated by dividing the estimated 8-month change by the pre-unwinding level (intercept) and multiplying by one hundred.

© 2025 Gupta S, et al. *JAMA Health Forum*.

eTable 1. Timeline of state Medicaid unwinding, rates and approaches to *automated* renewals, and state Medicaid Affordable Care Act (ACA) expansion status

Notes: Authors presentation of timing of Medicaid unwinding,[2] rates of automated, or ex parte, Medicaid eligibility renewals during unwinding (as of October 2023),[3] the number of data sources used to verify income for ex-parte renewals (as of May 2024),[4] and Medicaid Affordable Care Act (ACA) expansion status, by state,[5] from Kaiser Family Foundation's (KFF) Medicaid Enrollment and Unwinding Tracker. Unwinding start date is defined as the effective date of the first expected terminations. Information on the data sources used by Florida for income verification for ex parte not available. Data sources reported are in addition to Social Security Administration, Supplemental Nutrition Assistance Program (SNAP) and Temporary Assistance for Needy Families (TANF). Five states that expanded Medicaid under the ACA during the study period – South Dakota (7/1/2023), Nebraska (10/1/2020), Oklahoma (7/1/2021), Missouri (7/1/2021) and North Carolina (12/1/2023) – are excluded from the analysis.

State	State Medicaid Unwinding Start Date	Above median Share of Medicaid Renewals on *Ex parte* Basis (%)	Number of Data Sources States Used to Verify Income for *Ex parte* renewals	ACA Medicaid Expansion Status
Alabama	June 1, 2023	No (52.2)	4-5 Sources	No
Alaska	June 1, 2023	n/a	4-5 Sources	Yes
Arizona	April 1, 2023	Yes (88.9)	6-7 Sources	Yes
Arkansas	April 1, 2023	Yes (70.5)	4-5 Sources	Yes
California	July 1, 2023	Yes (63.5)	6-7 Sources	Yes
Colorado	June 1, 2023	No (46.9)	≤3 Sources	Yes
Connecticut	May 1, 2023	Yes (76.4)	≤3 Sources	Yes
Delaware	July 1, 2023	n/a	4-5 Sources	Yes
District of Columbia	June 1, 2023	Yes (68.7)	4-5 Sources	Yes
Florida	May 1, 2023	No (29.3)	n/a	No
Georgia	June 1, 2023	Yes (75.7)	4-5 Sources	No
Hawaii	June 1, 2023	Yes (76.5)	≤3 Sources	Yes
Idaho	April 1, 2023	No (28.5)	4-5 Sources	Yes
Illinois	July 1, 2023	Yes (65.6)	≤3 Sources	Yes
Indiana	May 1, 2023	Yes (71.7)	4-5 Sources	Yes
Iowa	May 1, 2023	No (54.2)	6-7 Sources	Yes
Kansas	May 1, 2023	No (32.4)	4-5 Sources	No
Kentucky	June 1, 2023	Yes (78.3)	6-7 Sources	Yes
Louisiana	July 1, 2023	Yes (79.7)	4-5 Sources	Yes
Maine	June 1, 2023	n/a	4-5 Sources	Yes
Maryland	June 1, 2023	Yes (74.9)	4-5 Sources	Yes
Massachusetts	June 1, 2023	Yes (86.3)	≤3 Sources	Yes
Michigan	July 1, 2023	No (57.1)	≤3 Sources	Yes
Minnesota	July 1, 2023	n/a	4-5 Sources	Yes
Mississippi	June 1, 2023	No (32.9)	4-5 Sources	No
Missouri	July 1, 2023	Yes (76.9)	4-5 Sources	During study period; excluded
Montana	June 1, 2023	No (40.2)	4-5 Sources	Yes
Nebraska	May 1, 2023	No (40.4)	6-7 Sources	During study period; excluded
Nevada	June 1, 2023	Yes (76.8)	4-5 Sources	Yes
New Hampshire	April 1, 2023	Yes (63.2)	6-7 Sources	Yes
New Jersey	June 1, 2023	n/a	4-5 Sources	Yes
New Mexico	May 1, 2023	Yes (75.9)	4-5 Sources	Yes
New York	July 1, 2023	No (36.8)	≤3 Sources	Yes
North Carolina	July 1, 2023	Yes (99.2)	≤3 Sources	During study period; excluded
North Dakota	June 1, 2023	No (43.5)	4-5 Sources	Yes
Ohio	May 1, 2023	No (56.6)	4-5 Sources	Yes
Oklahoma	May 1, 2023	No (32.6)	≤3 Sources	During study period; excluded
Oregon	October 1, 2023	n/a	4-5 Sources	Yes
Pennsylvania	May 1, 2023	No (11.6)	4-5 Sources	Yes

© 2025 Gupta S, et al. *JAMA Health Forum*.

Rhode Island	June 1, 2023	Yes (91.8)	≤3 Sources	Yes
South Carolina	June 1, 2023	No (44.9)	≤3 Sources	No
South Dakota	April 1, 2023	No (13.1)	4-5 Sources	During study period; excluded
Tennessee	June 1, 2023	Yes (62.9)	6-7 Sources	No
Texas	June 1, 2023	No (8.9)	4-5 Sources	No
Utah	May 1, 2023	No (53.5)	≤3 Sources	Yes
Vermont	June 1, 2023	Yes (68.3)	≤3 Sources	Yes
Virginia	May 1, 2023	Yes (75.4)	4-5 Sources	Yes
Washington	June 1, 2023	Yes (87.7)	6-7 Sources	Yes
West Virginia	May 1, 2023	No (21.8)	6-7 Sources	Yes
Wisconsin	June 1, 2023	No (20.4)	4-5 Sources	No
Wyoming	May 1, 2023	No (2.5)	≤3 Sources	No

© 2025 Gupta S, et al. *JAMA Health Forum*.

eTable 2. Study sample size by year and payer type.

Notes: Authors calculation using IQVIA Longitudinal Prescription (LRx) national retail pharmacy claims database from April 2020 to March 2024. Five states that expanded Medicaid under the ACA during the study period – South Dakota (7/1/2023), Nebraska (10/1/2020), Oklahoma (7/1/2021), Missouri (7/1/2021) and North Carolina (12/1/2023) – are excluded from the analysis. In any given year and throughout the study period, the combined percentage of adults with buprenorphine prescriptions across payer types may exceed 100%, as individuals can have multiple sources of coverage.

	Number of Adults with Buprenorphine	Percentage of Adults with Buprenorphine Prescriptions paid for by			
**YEAR**	**Prescriptions**	**Medicare**	**Medicaid**	**Commercial**	**Cash**
April 2020 - March 2021	1,210,940	11%	46%	47%	12%
April 2021 - March 2022	1,283,858	12%	47%	47%	11%
April 2022 - March 2023	1,320,835	12%	49%	45%	10%
April 2023 - March 2024	1,359,533	13%	49%	47%	11%
Overall (April 2020-March 2024)	2,405,970	12%	48%	46%	11%

eTable 3. Estimated change in Monthly Patients with Buprenorphine during the 3-year period prior to Medicaid unwinding by Payer Type

Notes: The estimated percent change in the monthly number of adults (age 18+) with filled buprenorphine prescriptions during the 3-year period prior to Medicaid unwinding (April 2020 to as much as September 2023) is presented. The analysis excludes five states that expanded Medicaid under the ACA during this period—South Dakota (7/1/2023), Nebraska (10/1/2020), Oklahoma (7/1/2021), Missouri (7/1/2021), and North Carolina (12/1/2023). Data are centered on the start of Medicaid unwinding in each state. The percent change in patients with fills during the pre-unwinding period was calculated by dividing the change in estimated monthly outcome over the 3-year pre-unwinding period (the month preceding unwinding relative to 36 months prior) by the estimated outcome at the start of the study period (36 months prior to unwinding in each state), then multiplying by 100.

State	All	Medicaid	Medicare	Commercial	Self-pay
Alabama	18	22	30	19	-7
Alaska	-6	-2	-3	-12	-14
Arizona	15	17	47	6	49
Arkansas	58	140	65	48	37
California	13	32	30	1	-37
Colorado	24	38	44	7	13
Connecticut	-1	-5	31	-4	-9
Delaware	18	20	20	13	16
District of Columbia	-15	-20	-10	-6	-29
Florida	12	110	18	6	-16
Georgia	22	24	24	24	5
Hawaii	-7	1	22	-20	-41
Idaho	28	64	30	5	-13
Illinois	8	11	25	3	-32
Indiana	25	60	24	-10	-40
Iowa	34	43	63	25	-17
Kansas	32	72	44	23	20
Kentucky	21	38	47	-18	-13
Louisiana	-1	16	13	-22	-36
Maine	7	19	13	-9	-25
Maryland	12	24	24	-7	-20
Massachusetts	-1	6	1	-12	2
Michigan	16	30	27	2	-45
Minnesota	28	37	51	18	-51
Mississippi	16	24	29	27	-11
Montana	18	17	46	11	16
Nevada	19	39	36	3	-8
New Hampshire	8	34	9	-8	-56
New Jersey	9	18	17	-1	-28
New Mexico	3	29	29	-46	53
New York	5	5	15	2	37
North Dakota	62	140	120	36	-12
Ohio	3	3	18	2	-19
Oregon	24	35	43	3	174
Pennsylvania	10	2	21	26	-40
Rhode Island	-5	-5	8	-12	20
South Carolina	17	21	36	14	6
Tennessee	11	103	34	-10	-32
Texas	20	46	42	13	29
Utah	21	93	34	-2	-11
Vermont	9	13	22	-3	-15
Virginia	49	123	52	3	-44
Washington	8	7	27	4	-12
West Virginia	11	22	34	1	-22
Wisconsin	16	27	38	0	-21
Wyoming	32	211	41	24	10

© 2025 Gupta S, et al. *JAMA Health Forum*.

eTable 4. State Changes in Monthly Number of Patients with filled Buprenorphine Prescriptions by payer type

Notes: An interrupted time series analysis, covering 36 months before and 8 months after the start of Medicaid unwinding (April 2020 to March 2024) in each state, was used to evaluate percent changes in the number of adults (age 18+) with filled buprenorphine prescriptions during the 8 months following the unwinding in each state. The analysis considered number of prescriptions by payer type, utilizing data from 45 U.S. states and Washington, D.C. Five states that expanded Medicaid during the study period—South Dakota (7/1/2023), Nebraska (10/1/2020), Oklahoma (7/1/2021), Missouri (7/1/2021), and North Carolina (12/1/2023)—were excluded. The percent change in patients with buprenorphine fills over 8 months was calculated by dividing the estimated 8-month change (sum of the post-unwinding intercept and eight times the post-unwinding monthly trend relative to the pre-unwinding intercept) by the pre-unwinding intercept, then multiplying by 100.

State	All	Medicare	Medicaid	Commercial	Self-Pay
Alabama	-4 (-6, -3)	-1 (-2, 1)	-23 (-26, -19)	-2 (-5, 1)	-2 (-12, 8)
Alaska	-3 (-10, 5)	15 (3, 27)	-15 (-22, -9)	20 (13, 27)	-5 (-55, 45)
Arizona	8 (5, 11)	2 (-1, 4)	10 (5, 15)	4 (1, 8)	52 (18, 86)
Arkansas	6 (4, 9)	12 (9, 14)	-1 (-5, 4)	1 (-2, 4)	48 (41, 56)
California	3 (0, 5)	7 (5, 10)	2 (0, 5)	1 (-2, 5)	6 (1, 12)
Colorado	2 (0, 4)	2 (0, 4)	-14 (-16, -12)	21 (17, 24)	47 (35, 60)
Connecticut	-2 (-3, -1)	2 (0, 5)	-10 (-12, -7)	6 (4, 8)	-7 (-23, 10)
Delaware	-4 (-7, -1)	-3 (-7, 0)	-17 (-21, -14)	16 (6, 27)	65 (41, 88)
District of Columbia	-11 (-16, -5)	-5 (-12, 3)	-22 (-30, -14)	6 (-11, 23)	32 (-1, 66)
Florida	-4 (-6, -2)	0 (-2, 2)	-39 (-45, -34)	4 (2, 6)	-8 (-12, -5)
Georgia	-1 (-2, 1)	10 (8, 12)	-33 (-37, -29)	3 (1, 5)	-3 (-8, 1)
Hawaii	8 (5, 12)	14 (8, 19)	2 (-2, 6)	14 (10, 19)	-20 (-65, 25)
Idaho	1 (0, 3)	11 (8, 14)	-22 (-25, -20)	21 (17, 24)	69 (56, 82)
Illinois	3 (1, 4)	7 (4, 10)	-10 (-13, -6)	13 (8, 19)	33 (17, 48)
Indiana	3 (2, 4)	5 (3, 8)	-13 (-15, -11)	34 (30, 38)	41 (-2, 84)
Iowa	8 (5, 11)	7 (1, 13)	-8 (-14, -3)	28 (21, 35)	3 (-30, 36)
Kansas	7 (5, 9)	16 (12, 20)	-4 (-8, 0)	8 (5, 11)	15 (4, 25)
Kentucky	-6 (-12, 1)	-2 (-4, 1)	-14 (-18, -10)	23 (-24, 70)	0 (-8, 7)
Louisiana	1 (-2, 4)	1 (-2, 3)	-11 (-15, -8)	24 (17, 32)	19 (5, 33)
Maine	3 (0, 6)	7 (4, 11)	2 (-1, 4)	6 (1, 10)	-16 (-30, -2)
Maryland	1 (0, 2)	5 (3, 7)	-3 (-4, -1)	7 (5, 8)	-1 (-8, 7)
Massachusetts	-2 (-5, 0)	-4 (-6, -2)	-7 (-20, 5)	5 (-16, 26)	32 (13, 50)
Michigan	0 (-1, 2)	-8 (-13, -3)	-10 (-12, -8)	15 (12, 18)	63 (54, 73)
Minnesota	3 (1, 6)	-2 (-6, 1)	0 (-3, 3)	6 (-1, 13)	113 (50, 176)
Mississippi	0 (-2, 2)	1 (-1, 3)	-22 (-28, -16)	3 (1, 5)	3 (-5, 11)
Montana	0 (-4, 3)	19 (13, 25)	-26 (-31, -22)	48 (44, 51)	108 (92, 124)
Nevada	7 (4, 9)	11 (7, 15)	-3 (-6, 1)	13 (9, 17)	24 (11, 38)
New Hampshire	-4 (-7, -2)	1 (-1, 3)	-25 (-41, -9)	16 (-7, 38)	67 (41, 93)
New Jersey	-2 (-3, 0)	4 (3, 5)	-12 (-14, -10)	8 (7, 10)	45 (35, 55)
New Mexico	2 (-1, 6)	3 (0, 6)	-15 (-29, -1)	57 (4, 109)	39 (16, 61)
New York	-6 (-7, -4)	2 (-1, 5)	-8 (-12, -3)	-8 (-17, 1)	34 (14, 54)
North Dakota	-8 (-13, -2)	12 (4, 20)	-27 (-33, -21)	4 (-1, 10)	10 (-11, 30)
Ohio	-5 (-10, 0)	0 (-1, 2)	-15 (-20, -9)	11 (0, 23)	4 (-8, 15)
Oregon	-5 (-10, 0)	6 (3, 9)	-11 (-19, -3)	-3 (-10, 4)	-10 (-43, 23)
Pennsylvania	-3 (-5, -1)	1 (-1, 2)	-17 (-23, -12)	10 (7, 13)	4 (-5, 14)
Rhode Island	-4 (-5, -2)	1 (-1, 3)	-18 (-20, -15)	14 (12, 16)	31 (-3, 65)
South Carolina	1 (-1, 4)	1 (-1, 4)	-21 (-27, -14)	9 (6, 12)	6 (-3, 15)
Tennessee	0 (-2, 2)	1 (-1, 3)	-11 (-13, -8)	5 (1, 10)	1 (-19, 21)
Texas	2 (0, 4)	18 (16, 21)	-47 (-50, -44)	2 (0, 4)	17 (1, 33)
Utah	-1 (-3, 1)	7 (3, 10)	-17 (-20, -14)	6 (4, 9)	9 (0, 18)
Vermont	-5 (-6, -3)	-5 (-7, -2)	-15 (-16, -13)	20 (17, 24)	8 (-4, 21)
Virginia	-1 (-4, 1)	0 (-2, 3)	-2 (-4, 0)	-2 (-11, 7)	22 (3, 40)
Washington	0 (-2, 1)	11 (8, 14)	-13 (-17, -10)	11 (8, 15)	12 (1, 24)
West Virginia	-4 (-7, -1)	-1 (-3, 2)	-11 (-15, -7)	21 (15, 27)	-16 (-25, -8)
Wisconsin	0 (-2, 1)	4 (3, 6)	-10 (-12, -8)	11 (8, 13)	38 (26, 49)
Wyoming	4 (1, 8)	15 (9, 21)	13 (2, 24)	2 (-2, 6)	0 (-14, 14)

© 2025 Gupta S, et al. *JAMA Health Forum*.

eTable 5. State Changes in Monthly Number of Patients with filled Buprenorphine Prescriptions by payer type and state policies that may support continued coverage for eligible individuals

Notes: An interrupted time series analysis, covering 36 months before and 8 months after the start of Medicaid unwinding (April 2020 to March 2024) in each state, was used to evaluate percent changes in the number of adults (age 18+) with filled buprenorphine prescriptions during the 8 months following the unwinding in each state. The analysis considered number of prescriptions by payer type, utilizing data from 45 U.S. states and Washington, D.C. Five states that expanded Medicaid during the study period—South Dakota (7/1/2023), Nebraska (10/1/2020), Oklahoma (7/1/2021), Missouri (7/1/2021), and North Carolina (12/1/2023)—were excluded. The percent change in patients with buprenorphine fills over 8 months was calculated by dividing the estimated 8-month change (sum of the post-unwinding intercept and eight times the post-unwinding monthly trend relative to the pre-unwinding intercept) by the pre-unwinding intercept, then multiplying by 100.

Sample	All	Medicare	Medicaid	Commercial	Self-Pay
All state sample (main analysis)	-2.89<br>(-3.95, -1.82)	0.34<br>(-0.84, 1.53)	-12.74<br>(-14.06, -11.42)	6.12<br>(3.74, 8.50)	7.24<br>(3.57, 10.91)
Share of automated renewals					
Above-median<br>(23 states)	-0.15<br>(-1.14, 0.85)	3.23<br>(2.38, 4.09)	-9.26<br>(-10.75, -7.77)	10.11<br>(5.69, 14.52)	12.73<br>(6.97, 18.49)
Below-median<br>(22 states)	-2.46<br>(-3.82, -1.11)	2.14<br>(1.06, 3.21)	-14.54<br>(-16.78, -12.30)	5.80<br>(3.70, 7.90)	4.27<br>(1.03, 7.51)
Number of income verification data sources used for automated renewals					
≤3 sources<br>(14 states)	-1.81<br>(-2.57, -1.05)	-0.73<br>(-2.05, 0.58)	-9.74<br>(-12.71, -6.78)	5.94<br>(1.20, 10.69)	24.07<br>(18.04, 30.11)
4-5 sources<br>(27 states)	-4.15<br>(-5.57, -2.73)	-0.67<br>(-2.47, 1.14)	-15.05<br>(-17.33, -12.77)	4.94<br>(3.05, 6.82)	10.53<br>(6.23, 14.83)
6-7 sources<br>(9 states)	-1.26<br>(-3.24, 0.72)	3.40<br>(2.08, 4.73)	-9.94<br>(-11.88, -8.00)	9.90<br>(2.55, 17.25)	-0.10<br>(-5.69, 5.49)
Pre-pandemic ACA Medicaid expansion status					
ACA expansion<br>(36 states)	-3.40<br>(-4.56, -2.23)	-1.05<br>(-2.42, 0.31)	-11.93<br>(-13.29, -10.57)	7.47<br>(4.34, 10.59)	12.70<br>(8.96, 16.45)
ACA non-expansion<br>(10 states)	-1.11<br>(-2.29, 0.07)	4.65<br>(3.40, 5.91)	-20.56<br>(-22.52, -18.60)	3.47<br>(1.82, 5.11)	0.57<br>(-4.66, 5.79)

© 2025 Gupta S, et al. *JAMA Health Forum*.

eFigure 1. Changes in Monthly Number of Patients with filled Buprenorphine Prescriptions, overall and prescription fills paid for by Medicaid

Notes: An interrupted time series analysis, covering 36 months before and 8 months after the start of Medicaid unwinding (April 2020 to March 2024) in each state, was used to evaluate percent changes in the number of adults (age 18+) with filled buprenorphine prescriptions during the 8 months following the unwinding in each state. The analysis considered both the overall number of prescriptions and those paid for by Medicaid, utilizing data from 45 U.S. states and Washington, D.C. Five states that expanded Medicaid during the study period—South Dakota (7/1/2023), Nebraska (10/1/2020), Oklahoma (7/1/2021), Missouri (7/1/2021), and North Carolina (12/1/2023)—were excluded. The percent change in patients with buprenorphine fills over 8 months was calculated by dividing the estimated 8-month change (sum of the post-unwinding intercept and eight times the post-unwinding monthly trend relative to the pre-unwinding intercept) by the pre-unwinding intercept, then multiplying by 100.

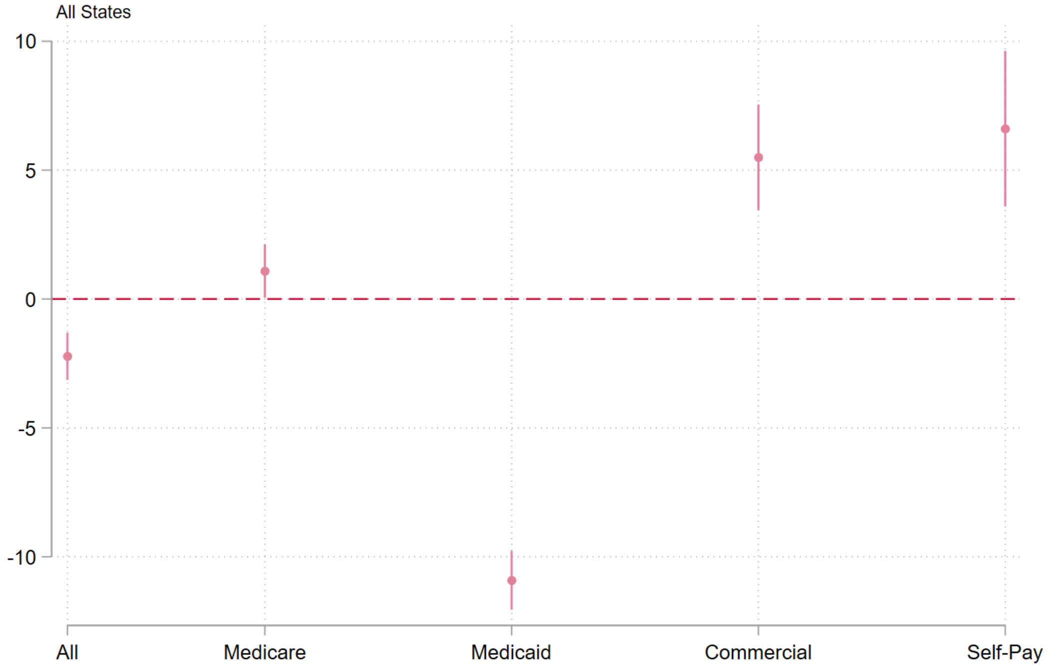

© 2025 Gupta S, et al. *JAMA Health Forum*.

eFigure 2. Estimated change in Monthly Patients with Buprenorphine Prescription Fills during the 3-year period prior to Medicaid unwinding by Payer Type

Notes: The estimated percent change in the monthly number of adults (age 18+) with filled buprenorphine prescriptions during the 3-year period prior to Medicaid unwinding (April 2020 to as much as September 2023) is presented. The analysis excludes five states that expanded Medicaid under the ACA during this period—South Dakota (7/1/2023), Nebraska (10/1/2020), Oklahoma (7/1/2021), Missouri (7/1/2021), and North Carolina (12/1/2023). Data is centered on the start of Medicaid unwinding in each state. The percent change in patients with fills during the pre-unwinding period was calculated by dividing the change in estimated monthly outcome over the 3-year pre-unwinding period (the month preceding unwinding relative to 36 months prior) by the estimated outcome at the start of the study period (36 months prior to unwinding in each state), then multiplying by 100.

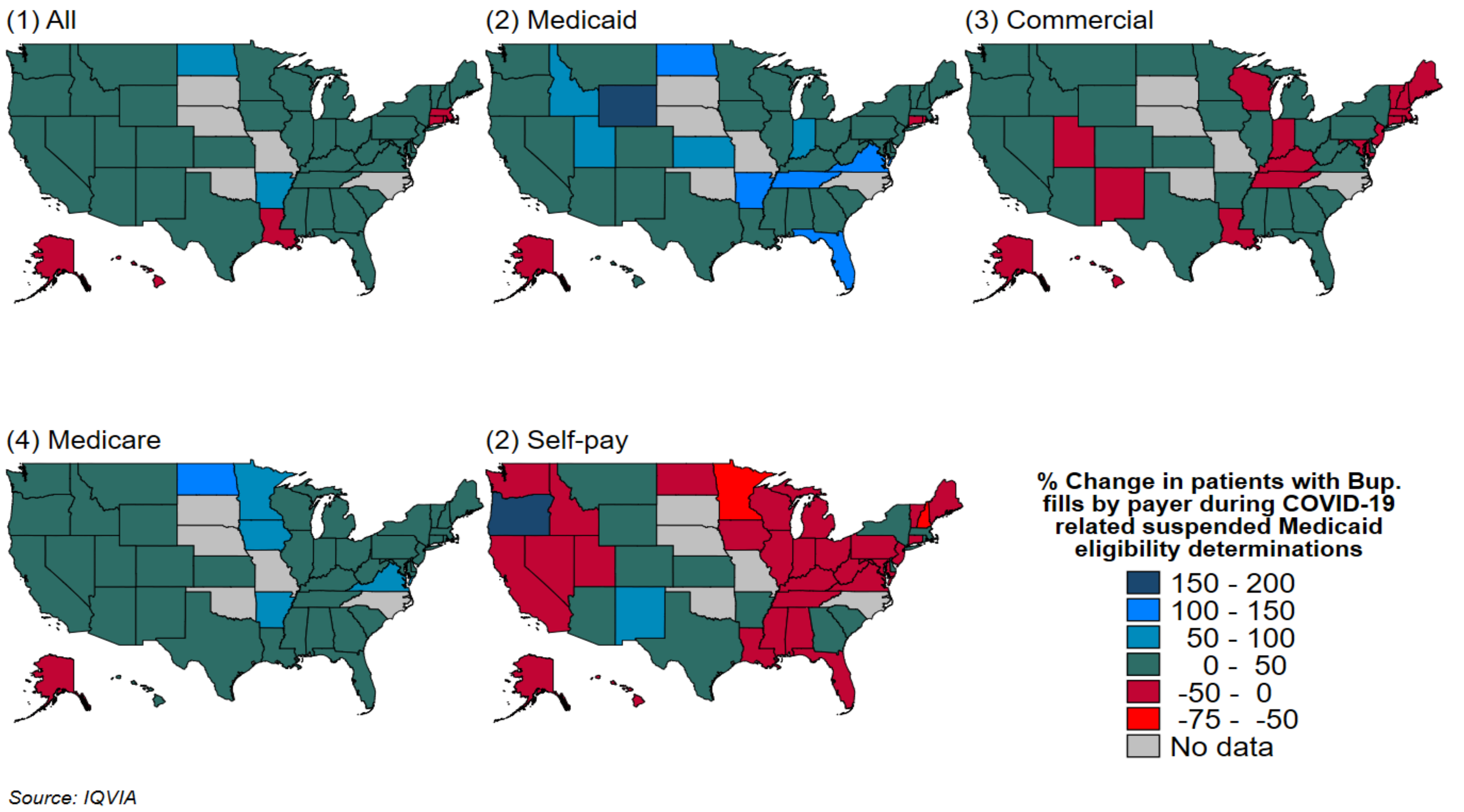

© 2025 Gupta S, et al. *JAMA Health Forum*.

REFERENCES

[1] Bureau UC. State Population Totals and Components of Change: 2020-2024. Census.gov. Accessed January 5, 2025. https://www.census.gov/data/tables/time-series/demo/popest/2020s-state-total.html

[2] Medicaid enrollment and unwinding tracker - federal unwinding and enrollment data. KFF; 2024. Available from: https://www.kff.org/report-section/medicaid-enrollment-and-unwinding-tracker-national-federal-unwinding-and-enrollment-data/

[3] Understanding Medicaid Ex Parte Renewals During the Unwinding | KFF; 2024. Available from: https://www.kff.org/policy-watch/understanding-medicaid-ex-parte-renewals-during-the-unwinding/

[4] A Look at Medicaid and CHIP Eligibility, Enrollment, and Renewal Policies During the Unwinding of Continuous Enrollment and Beyond - Report - 10404 | KFF; 2024. Available from: https://www.kff.org/report-section/a-look-at-medicaid-and-chip-eligibility-enrollment-and-renewal-policies-during-the-unwinding-of-continuous-enrollment-and-beyond-report/

[5] Status of State Medicaid Expansion Decisions | KFF; 2024. Available from: https://www.kff.org/status-of-state-medicaid-expansion-decisions/

© 2025 Gupta S, et al. *JAMA Health Forum*.
