# Supplementary material for: Trends in Access to Medications for Opioid Use Disorder
Source: JAMA Health Forum. 2025 Apr 4;6(4):e250393. doi: 10.1001/jamahealthforum.2025.0393 (PMC11971676; doi:10.1001/jamahealthforum.2025.0393)
Supplement: Supplement 2. — Data Sharing Statement [file jamahealthforum-e250393-s002.pdf]

**Data Sharing Statement**

**Data**
**Data available:** No

**Additional Information**
**Explanation for why data not available:** The data use agreement of the proprietary data used for this study does not allow us to submit the data.
